# On chip random lasing performance of the acceptor dye in a specially designed linear and zig zag array of microdroplets with intrinsic disorder

**DOI:** 10.1038/s41598-022-07104-8

**Published:** 2022-03-10

**Authors:** Aswathy Sundaresan, Soniya Nuchikkat, K. Shadak Alee

**Affiliations:** 1 School of Physics, IISER - Thiruvananthapuram, Maruthamala PO, Vithura, Thiruvananthapuram, Kerala 695551 India; 2 Department of Physics and Nanotechnology, SRM Institute of Science and Technology, Kattankulathur, Chengalpattu (District), Tamil Nadu 603203 India

**Keywords:** Optofluidics, Micro-optics, Lasers, LEDs and light sources

## Abstract

We demonstrate high-quality on chip random lasing of the acceptor dye using a specially designed dynamic linear array of microdroplets with unconventional shapes that do not support the whispering gallery modes. The intrinsic disorder in the droplet array consequent to its dynamic nature is utilized in achieving the randomness in the lasing emission without deliberately adding the disorder as in ordinary random laser systems. The novelty of the generated structure is illustrated by comparing its emission characteristics with two other arrays made of microspheres and microtriangular droplets arranged in a zig zag fashion. We show that only the linear array of microdroplets supports lasing in the acceptor emission. The design of the array structure allows to further reduce the lasing threshold drastically either by incorporating the radiative energy transfer or by tuning the number of acceptor dye molecules. The highly directional, well-controlled, and intense emission of weakly emitting dye generated on chip without the aid of conventional methods opens up a new scheme of random lasing generation mediated by microdroplets.

## Introduction

Lasing in the tiny microdroplets were so fascinating ever since the demonstration of whispering gallery modes (WGMs) back in 1984 by Chang et al.^1^ These morphology dependent resonances are characterized with ultra-high-quality lasing modes, which makes the microresonators a supreme contender in delivering efficient lasing emission for various potential applications^2^. Later, Whiteside’s discovery of microfluidic devices based on soft lithography technology synthesised unique functionalities by combining optics and fluidics in a synergistic way^3,4^. This eventually led to the generation of microdroplets on microfluidic platforms^5–11^ and, as a result, the development of droplet dye laser systems for lab-on-a-chip^12–16^. Owing to the excellent controllability over the size and shapes of the microdroplets, the microfluidic systems serve as an exceptionally adequate platform to generate and investigate the lasing behaviour of the array of microdroplets with unconventional shapes. When an individual resonator lases in WGMs, an array of resonators can essentially deliver the collective lasing pertaining to the structure of the array. Ref.^17^ has demonstrated such collective behaviour of a linear array of randomly separated amplifying droplet microcavities generated in air. Later they investigated the origin and spectral features of the collective lasing modes in the vantage point of random lasing by introducing controlled disorder in the inter-droplet separation and size^18^. A random laser, unlike the ordinary lasers, relies on scattering-induced feedback in the presence of gain, with the scattering centres essentially acting as refractive index irregularities in the system under investigation^19–23^. In contrast to the conventional random laser systems, the generated droplet arrays in ref^18,24^ with non-uniformity in the refractive index along the array axis manifest structural disorder based random lasing. The resultant structures are fundamentally treated as 1D scattering systems with an induced disorder to demonstrate the random lasing transpired in the system. The random lasing in these systems are identified essentially as the collective modes pertaining to the structure, arising from the states introduced into the stopgaps at the given disorder strength. Similar studies are reported earlier using micron-sized glass slides also^25^. In the domain of microdroplets, as the individual entities support WGMs and the array is characterized with coupled cavity modes, the available gain will be shared between the two. But the high quality and small mode volume of the WGMs makes them superior in the competition. So the random lasing performance of the droplet arrays is hardly investigated yet. Hence either decoupling the WGMs and cavity modes or eliminating the WGMs is preferable to selectively channelize the gain in the system-cavity modes and to investigate the vulnerability of microdroplets towards delivering pure random lasing. An investigation in this aspect is performed in microdiscs using finite difference time domain (FDTD) method and the results are given in Supplement 1. In addition, to date, the lasing behaviour studies of microdroplets with unique shapes other than spherical remains unexplored.

Consequent to the non-abundance of pump wavelengths, demonstrations that illustrate the efficient excitation and emission of organic dye molecules through indirect excitation mechanisms are always demanding. This offers excellent feasibility to realize efficient lasing in the entire visible range using various donor–acceptor pairs. Here, for the first time using the microfluidic platform, we are demonstrating the random lasing in the acceptor emission and further reduction in the lasing threshold utilizing the radiative energy transfer (RET) in a dynamic 1D array of microdroplets with intrinsic disorders in the sizes and inter-droplet separation. The generated droplets lack the existence of WGMs and is made possible by selectively choosing the refractive index of the droplet and the background medium. Also, the shape of microdroplets constituting the array is distinctive from the conventional lasing droplets^12–17,26^. This, along with the design of the array helps in directional propagation of the light and hence the initialization of the system-cavity modes. The intrinsic disorder of the generated droplets in the presence of gain is employed in triggering random lasing in the droplet array. The unit cell of the linear array we developed consists of a symmetric passive and asymmetric active droplet separated by a thin layer of mineral oil. Selectively, the asymmetric droplets in the array are doped with an acceptor dye known to have minimal pump absorption. Hence the lasing behaviour of these dyes under the given conditions is a direct measure of the efficacy of the droplet array. In addition, this array of microdroplets is designed in such a way to reduce the acceptor lasing threshold in two ways: either by tuning the size of the acceptor doped droplets or by introducing donor molecules in the position of passive droplets, thereby incorporating the RET from the donor to the acceptor. The performance of the linear array is evaluated also by comparing the acceptor emission with two other arrays made of microspheres and microtriangular droplets arranged in a zig zag fashion. The generation of highly stable microdroplets of unique shapes deviating from the conventional spherical droplets, and their practical usage in demonstrating the random lasing is an advancement in droplet microfluidics. Besides, this platform offers the easiest channel to switch on and switch off the well-explored WGM lasing simply through the selection of different solvents and the background medium.

## Experimental results and discussion

The linear, microsphere and microtriangular droplet arrays are generated using a microfluidic assembly consisting of microfluidic pumps, microfluidic chip and a high-speed digital microscope purchased from Dolomite Microfluidics. The microfluidic chip consists a 190 µm deep and 390 µm wide Polydimethylsiloxane (PDMS, n_PDMS_ = 1.403) channel. PDMS is a mineral-organic polymer of the siloxane family, widely used for the fabrication of microfluidic devices. The siloxane bonds in PDMS offer them high level of viscoelasticity. In addition to this, other properties such as optical transparency, low autofluorescence, low cost, low interfacial free energy, good thermal stability, isotropic and homogeneous nature etc. makes them ideal for the microfluidic applications. PDMS can be easily bonded with glass through plasma treatment, for the production of microfluidic chip^3^. A detailed description regarding the generation of microdroplets using the microfluidic chip is given in Fig. S1 of Supplement 2. The solvent and the background medium are selected so that the generated droplets do not support WGMs, whereas the channel of the chip acts as a guiding layer. Accordingly, ethylene glycol (EG, n_EG_ = 1.43) is used to dissolve the dyes and mineral oil (MO, n_MO_ = 1.467) is used as the background medium. Rhodamine B (RB) and Nile blue (NB), at a concentration of 2.5 mM are used as the donor and acceptor dyes respectively. The imaging of the droplets under white light illumination is done using high-speed digital microscope exclusively designed for microfluidics. The microscope is equipped with a high power LED for the illumination of the droplets and a colour camera with CMOS sensor for the imaging. The generated array configurations are excited using a Nd-YAG laser of 6 ns duration, emitting at 532 nm, and the imaging of the droplets under pump illumination is done using a colour CCD. A schematic of the experimental setup is given in Fig. S2 of Supplement 2.

**Figure 1:**
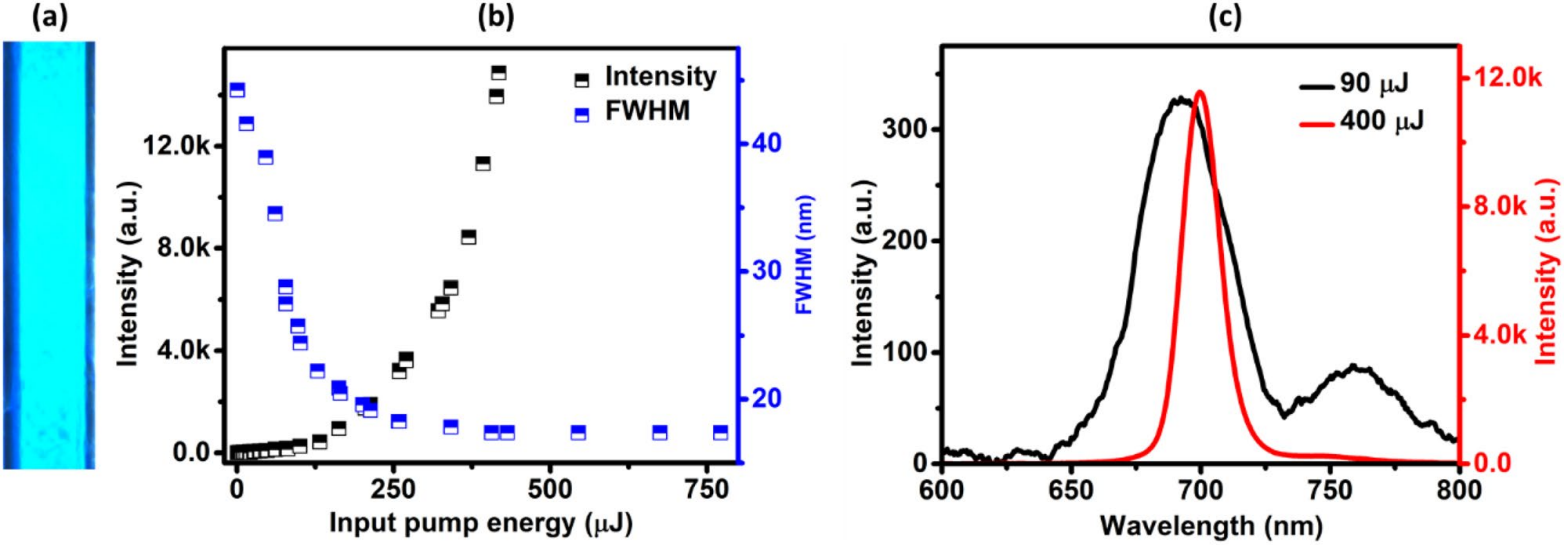
Image and emission characteristics of the continuous flow of dye solution. (**a**) Microscopic image of the continuous flow of acceptor dye solution through the channel of the chip. (**b**) Variation in the emission intensity and FWHM with input pump energy, collected from a continuous flow of acceptor dye solution. (**c**) A comparison of the emission spectral profile at low (black) and high (red) pump energies corresponding to 90 μJ and 400 μJ respectively.

Prior to moving to the microdroplet generation, we have analysed the performance of the acceptor dye in terms of the emission intensity and full width at half maximum (FWHM) at the given pump wavelength in a continuous flow of dye solution through the channel of the chip. An image of the generated flow is shown in Fig. 1a. When the input pump energy is gradually increased, the emission intensity has shown a linear rise up to a pump energy of ~ 130 μJ and thereafter, a dramatic rise indicating the onset of amplified spontaneous emission (ASE), as can be seen from Fig. 1b. The FWHM, on the other hand, showed a gradual reduction and realized a minimum bandwidth of only 17 nm as can also be seen from Fig. 1b. A comparison of the emission spectra at low (90 μJ) and high (400 μJ) pump energies with respect to the ASE threshold (130 μJ) is shown in Fig. 1c. It can be seen from Fig. 1b,c that the emission from the continuous flow is restricted only to the ASE regime consequent to the non-availability of a cavity.

**Figure 2:**
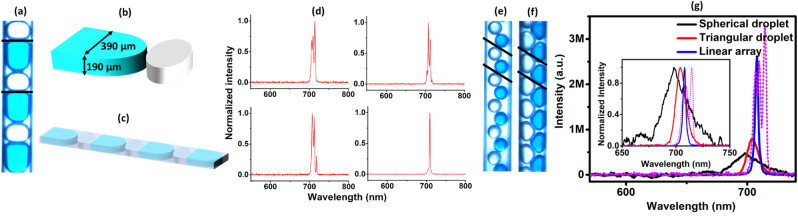
Image and emission characteristics of the linear and zig zag array of droplets. (**a**) Linear dynamic droplet array generated to achieve the acceptor lasing. (**b**) 3D view of the unitcell of the linear array (**c**) Schematic of the microdroplets flowing through the channel of the chip (**d**) The emission from the acceptor molecules in a linear droplet array for different pump pulses at an energy of ~ 130 μJ, in the absence of donor. Zig zag droplet arrays of (**e**) spherical and (**f**) triangular microdroplets generated to compare the emission from the linear array. (**g**) A comparison of the emissions from the spherical (black), triangular (red) and linear (blue) droplet arrays in the absence of donor at an input energy of ~ 1 mJ. The intensity of emission from the spherical droplet array is scaled by a factor of 2000 and that from the triangular droplet array is scaled by a factor of 100. The spectrum in dotted magenta lines represents the emission from the linear array for another pulse at the same pump energy. The inset represents the respective normalized emissions.

Interestingly, when the continuous flow of dye solution is replaced with our specially designed dynamic microdroplets, we have observed monumental changes in the emission spectra. In microfluidics, an experimental demonstration of any dynamic array of microdroplets is always characterized with random fluctuations in their sizes and inter-droplet separation. This can be due to many factors constituting the generation of droplets like the minute fluctuations in the pump pressure, flow rate etc. A dynamic array with all microdroplets of equal sizes and constant inter-droplet separation is only an ideal condition. If the disorder is not given intentionally, the resultant fluctuations are called intrinsic disorders, as they are unavoidable and are an inherent nature of the system. Since the droplet parameters like the size and inter-droplet separation are instantly varying as it flow through the channel of the chip, the resultant configuration of the droplet array will be different each time. Figure 2a in the manuscript represents one such an image of the dynamic linear array of droplet characterized with a size and inter-droplet separation unique to the instant of recording of the image. The unit cell defined by the droplets within the parallel lines consists of an asymmetric active droplet of NB in EG (blue colour, with approximate dimension 435 × 390 × 190 μm^3^), adjacent to a passive droplet of EG (white colour, with approximate dimension 250 × 390 × 190 μm^3^), separated by a thin layer of MO of approximate thickness 1 μm. The layer of oil essentially prevents the coalescence of droplets and allows only the radiative modes to couple to the nearby droplets. The aforementioned droplet dimensions are calculated by performing a pixel to length conversion calculation since the droplet imaging is done using a CMOS sensor associated with the high-speed digital microscope. As the channel dimensions are known, the pixel equivalent of unit length can be calculated, enabling the precise measurement of droplet sizes. A 3D view of the unit cell is shown in Fig. 2b and a schematic of the microdroplets flowing through the channel of the chip is shown in Fig. 2c. Consequent to the continuous random fluctuations in the droplet parameters, the pump pulses are experiencing different array configurations each time during its arrival. The emission from the linear droplet array for different pump pulses at energy of ~ 130 μJ is shown in Fig. 2d. The spectrometer used for recording the emission from the droplet array is configured in the trigger mode with the pump pulses as the trigger. Simultaneously, the energy of individual trigger pulses is also saved. This offers the feasibility to have the spectral data corresponding to each pulse of known energy. Four spectra corresponding to pump pulse energy of ~ 130 μJ are selected and shown in Fig. 2d. Surprisingly, in contrast to the emission behavior of the continuous flow of dye solution as shown in Fig. 1c, the emission spectra from the generated array of droplets are characterized with multiple emission peaks having ~ 2.5 nm bandwidth and are distinct from pulse to pulse. The observed bandwidth of the emission is limited by the low resolution of the spectrometer we employed, as also reported in ref.^27^. At input pump energy of ~ 130 μJ, the emission from the continuous flow has entered only the ASE regime. Whereas at this energy, the linear array of microdroplets are already delivering the lasing emission. In addition, the flexibility of the generated droplet system to introduce the acceptor dye only to the asymmetric droplets offers a chance of further introduction of a donor dye to the passive ones if required. This can have great impact on reducing the acceptor emission threshold through the RET.

Now, in order to confirm whether the spectral behaviour characterized with multiple peaks is unique to the linear array, we have generated microsphere (radius = 120 ± 1 μm) and microtriangular (edge length = 250 ± 2 μm) droplet arrays as well, as shown in Fig. 2e,f respectively. The unit cell of these droplet arrays consists of an active spherical (triangular) droplet of NB and a passive droplet of EG placed at an angle, such that the entire array forms into an arrangement of droplets in the zig zag fashion. Respective unit cells are shown by the droplets within the parallel lines. Note that the voids in all the arrays are filled with MO. Figure 2g is a comparison of the emission from the linear (blue), spherical (black) and triangular (red) droplet arrays at an input pump energy of ~ 1 mJ. This high energy is chosen to compare the spectra since the spherical droplet array has started giving emission at this pump energy only. The spectrum in dotted magenta lines represents the emission from the linear array for another pump pulse at the same pump energy. In the figure, the intensity of emission from the spherical droplet array is scaled by a factor of 2000 and that from the triangular droplet array is scaled by a factor of 100. The inset represents the respective normalized emission spectral profile. As can be seen from the figure, both zig zag droplet arrays are delivering single peak emission with a broad spectral bandwidth similar to the emission from a continuous flow as shown in Fig. 1c. Clearly, at the given pump energy, only the linear array is showing a unique emission performance.

**Figure 3:**
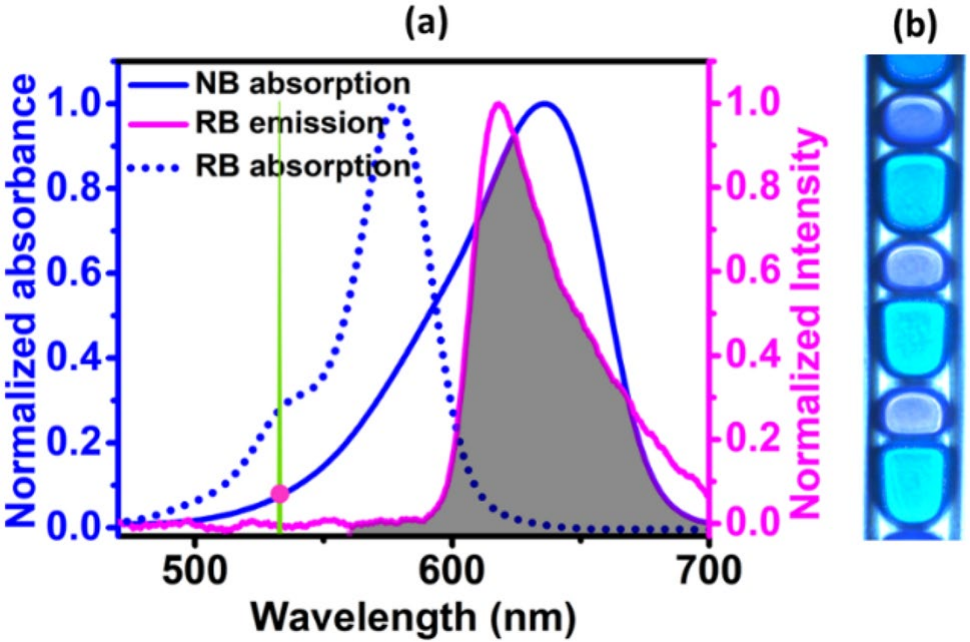
RET condition and the microdroplet generated to demonstrate it. (**a**) Spectral overlap (shaded region) between the donor fluorescence (magenta) and acceptor absorption (solid blue lines), along with the pump absorption by the acceptor molecules (represented by the magenta circle). The spectrum in dotted blue lines represents the donor absorption spectrum. (**b**) Linear droplet array made by replacing the passive EG droplets with donor doped EG.

**Figure 4:**
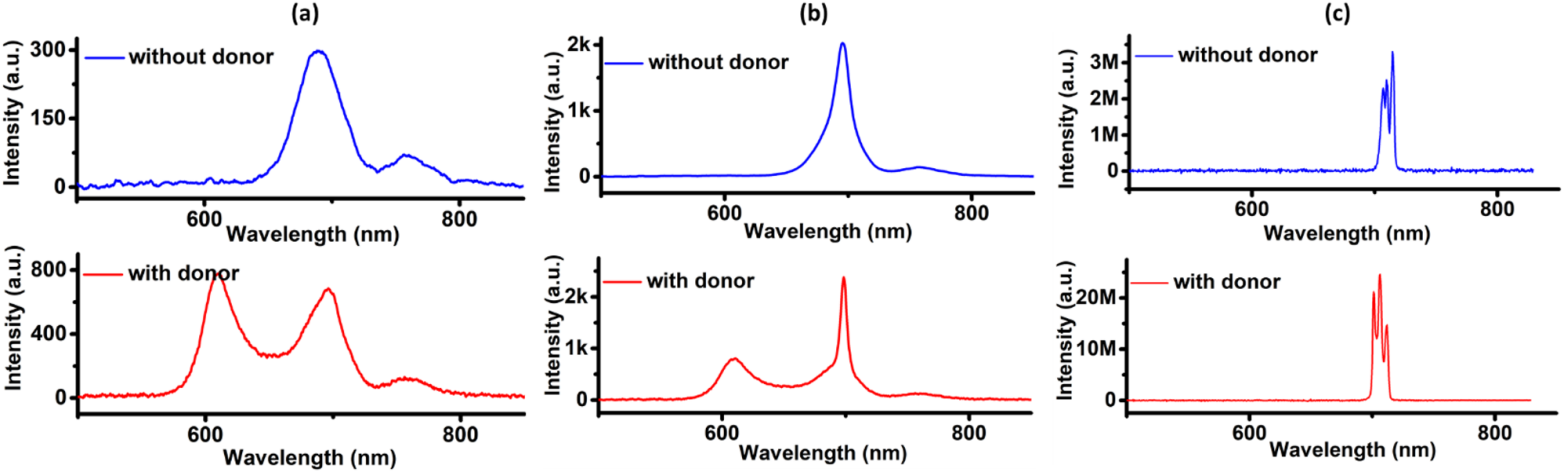
A comparison of the emission spectral profile from the linear array of droplets before (blue) and after (red) introducing the donor molecules at (**a**) low (**b**) intermediate and (**c**) high pump energies.

After examining the emission behaviour of the linear, spherical and triangular droplet arrays, we are proposing two distinct strategies to reduce the random lasing threshold of the acceptor emission in the linear droplet array. First, all the passive EG droplets in the array can be replaced with donor doped EG to utilize the RET from the donor to the acceptor. Second, keeping the passive EG droplets of the linear array in position, the length of the NB doped asymmetric droplets can be tuned to increase the number of acceptor dye molecules. The spectral overlap (shaded area) between the donor fluorescence (magenta) and acceptor absorption (solid blue lines) as demanded by the RET is shown in Fig. 3a. The figure also manifests a negligible pump absorption by the acceptor molecules through the coincidence of the pump spectrum (green) and the tail region of the acceptor absorption as represented by the magenta circle. The absorption spectrum of the donor (dotted blue lines) is also provided for a comparison of the pump absorption by the donor and acceptor molecules. The resultant droplet array after replacing the passive EG droplets with RB doped EG are shown in Fig. 3b. The introduction of donor molecules has changed the acceptor emission dramatically. A comparison of the emission spectral profile from the linear array of droplets at low, intermediate and high pump energies, before (blue) and after (red) introducing the donor molecules are shown in Fig. 4a–c respectively. At low pump energies, the acceptor emission is characterized with a broad emission spectrum in the absence of a donor, as presented in Fig. 4a. When the donor molecules are included in the linear droplet array, the spectrum is characterized with two dominant peaks each representing the donor and acceptor emissions, as can be seen from the figure. The RET from the donor to the acceptor is weak at these levels of pump energies. Gradual increment in the input pump energy results in the shrinking of acceptor emission bandwidth even in the absence of a donor. This is designated by the nature of the spectral curve at the intermediate pump energies as in Fig. 4b and it occurs when the acceptor emission enters the ASE regime. In the presence of donor, the RET is substantial at the intermediate pump energies. This leads to fast growth in the acceptor emission intensity with reduced bandwidth as can also be seen from Fig. 4b. At high pump energies, the emission spectrum is characterised with multiple lasing peaks as presented in Fig. 4c and the RET is very significant that the donor emission is completely absent. The acceptor emission arising in the presence of donor has shown ~ 8 times enhancement in the intensity compared to that in the absence of donor molecules. The introduction of donor molecules in this way to the linear array allows the complete radiative transfer of donor emission to the acceptor molecules. Since the size of donor doped droplets is made smaller than the acceptor doped asymmetric droplets, the chance of any remnant donor emission from the droplet array is hindered. In addition, the interaction length provided by the droplet array is much higher, resulting in complete reabsorption of the donor emission by the acceptor molecules and hence a pure acceptor lasing emission.

**Figure 5:**
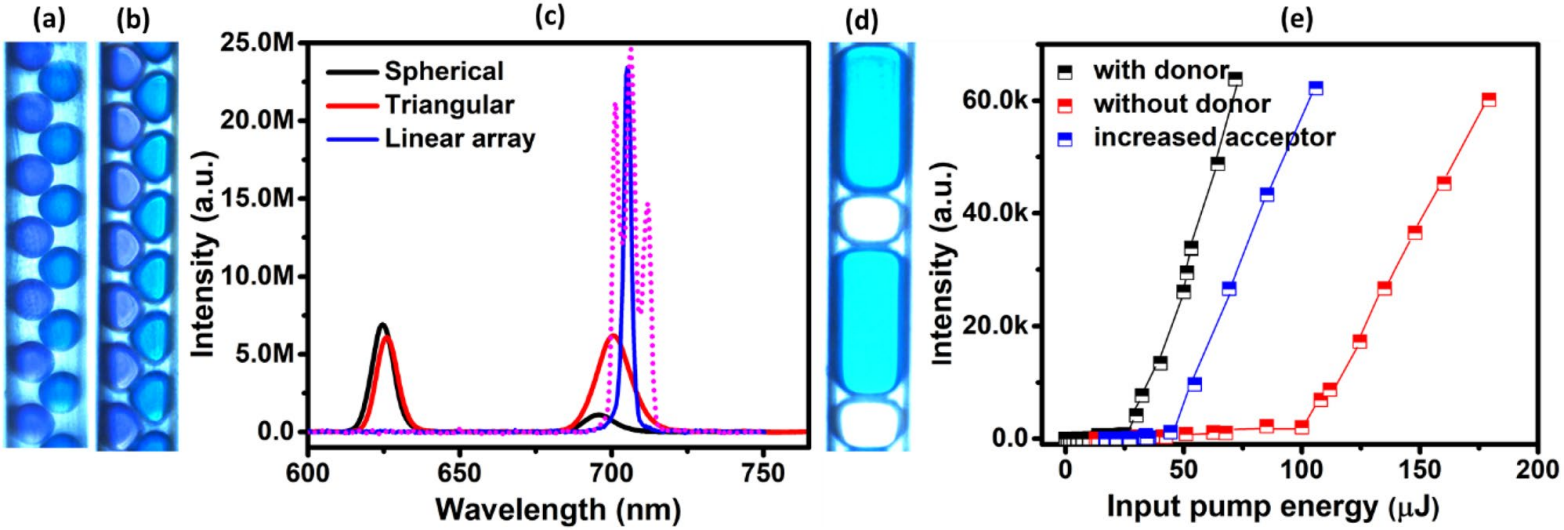
Droplet arrays made by replacing the passive EG droplet with donor doped EG in (**a**) spherical and (**b**) triangular droplet arrays. (**c**) A comparison of the emissions from the spherical (black), triangular (red) and linear (blue) droplet arrays in the presence of donor for pump pulses at input energy of 1 mJ. The spectrum in dotted magenta lines represents the emission from the linear array for another pulse at the same pump energy. (**d**) Droplet array generated with an increased size of the acceptor doped droplet. (**e**) Threshold behavior of the linear droplet array when the donor is absent (red), in the presence of donor (black) and when the size of the acceptor droplet is increased in the absence of donor (blue).

To analyze the performance of the spherical and triangular droplet arrays in the presence of RET, we have replaced the passive droplets in the respective droplet arrays with the corresponding donor counterpart as depicted by Fig. 5a,b respectively. A spectral comparison between the pure acceptor lasing from the linear droplet array (blue) and the emissions from the zig zag droplet arrays (black and red) at input energy of ~ 1 mJ are shown in Fig. 5c. Both zig zag droplet arrays have shown dual peak emission as expected, with dominating donor emission intensity in the spherical droplet array and comparable donor and acceptor intensities in the triangular droplet array, as can be seen from the figure. The spectrum in dotted magenta lines represents the emission from the linear array for another pump pulse at the same energy. The spectra indicate an inefficient or partial radiative energy transfer from the donor molecules to the acceptor in the zig zag arrays since these structures do not provide any high-quality modes. As mentioned earlier, the linear array is designed in a way to accommodate more number of acceptor molecules than the donor molecules, reducing the chance of any remnant donor emission. On the contrary, the donor and acceptor droplets in the microsphere as well as microtriangular arrays are characterized with comparable size, so is the number of donor and acceptor molecules. This and the absence of complete RET could lead to the appearance of donor peak in the emission spectrum as can be seen from Fig. 5c. Forster resonance energy transfer (FRET) has also been considered as an efficient way to reduce the acceptor lasing threshold by keeping the donor molecules in close proximity to the acceptor and thereby triggering the non-radiative energy transfer from the donor to the acceptor^28–30^. But this, in microfluidics, demands the mixing of donor and acceptor dye solutions where the solubility of different laser dyes in solvents can be a barrier. The generated microdroplet arrays, on the other hand, offer excellent feasibility to selectively dope the donor and acceptor dye molecules in the desired solvents to effectively utilize the RET instead of FRET towards reducing the acceptor lasing threshold.

To illustrate the second strategy with the aim of reducing the acceptor lasing threshold, we have tuned the size of the asymmetric droplets as shown in Fig. 5d without disturbing the passive droplets of EG. The length of the acceptor droplet is increased to ~ 950 μm from the initial length of ~ 435 μm, keeping the width the same. This has effectively increased the number of acceptor dye molecules available and reduced the lasing threshold by providing additional gain. The reduced lasing thresholds are compared with the actual threshold obtained when none of the threshold reducing strategies are employed. The respective threshold comparing curves are shown in Fig. 5e. It can be seen from the figure that replacing the passive droplets made of EG with the donor doped EG droplets is more effective in reducing the lasing threshold than increasing the size of the acceptor droplet. The former has reduced the lasing threshold from ~ 100 μJ to ~ 28 μJ and the latter to ~ 49 μJ. This is because the design of the array allows a dramatic rise in the number of excitation photons available for the acceptor consequent to the complete radiative transfer from the donor.

## Simulation results and discussion

**Figure 6:**
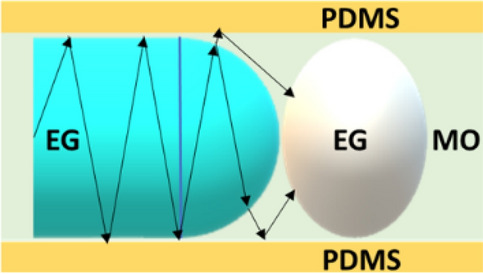
Schematic of light propagation within the unitcell of linear array of microdroplets.

The origin of lasing in the linear array of droplets relies on a couple of factors including the array structure, the size of the droplets, the refractive indices of the droplets and the background and so on. A schematic of the unit cell of the linear array is shown in Fig. 6. In 2D, the asymmetric droplet made of EG (blue) can be well approximated to the union of a rectangle and a half-circle. As shown in figure, the rectangle is surrounded by the PDMS layer, whereas the MO surrounds the half-circle. As n_EG_ > n_PDMS,_ throughout the rectangular region light sees a rarer medium as the immediate background, undergoing internal reflection back to the channel's core. Thus this region acts as a supporting stage for light propagation. The emission escaping from the circular top can also revert to the channel when it finds the PDMS layer as shown in the figure. Thus the shape of individual droplets, the selection of the solvent as well as the structure of the array are helping with the localization and longitudinal guidance of the isotropic emission from the dye molecules within the channel itself. Consequent to the light guiding ability of the droplet array, the initial fluorescence generated in all directions will be propagated along the longitudinal direction and experiences gain in multiple droplets and gets amplified. Such propagating light faces the variation in refractive indices along the axis due to the presence of thin layers of mineral oil between the droplets. These interfaces essentially provide the feedback for longitudinal propagation through multiple reflections and transmissions of light. Hence the dynamic droplet array is identical to a 1D photonic crystal with a disorder in the sizes and spacing between the constituent layers, as mentioned previously. Such structures are known to exhibit perturbed modes, which are highly sensitive to the disorder strength^18,31,32^. Under optical gain, these modes realize amplification and emerge as lasing modes, which is supported by the multiple lasing peaks observed in the acceptor emission profile, as shown in Fig. 2d. This collective emission from the array, which is a cooperative contribution of each droplet relies on the Fabry–Perot resonances and is highly directional along the channel of the chip.

**Figure 7:**
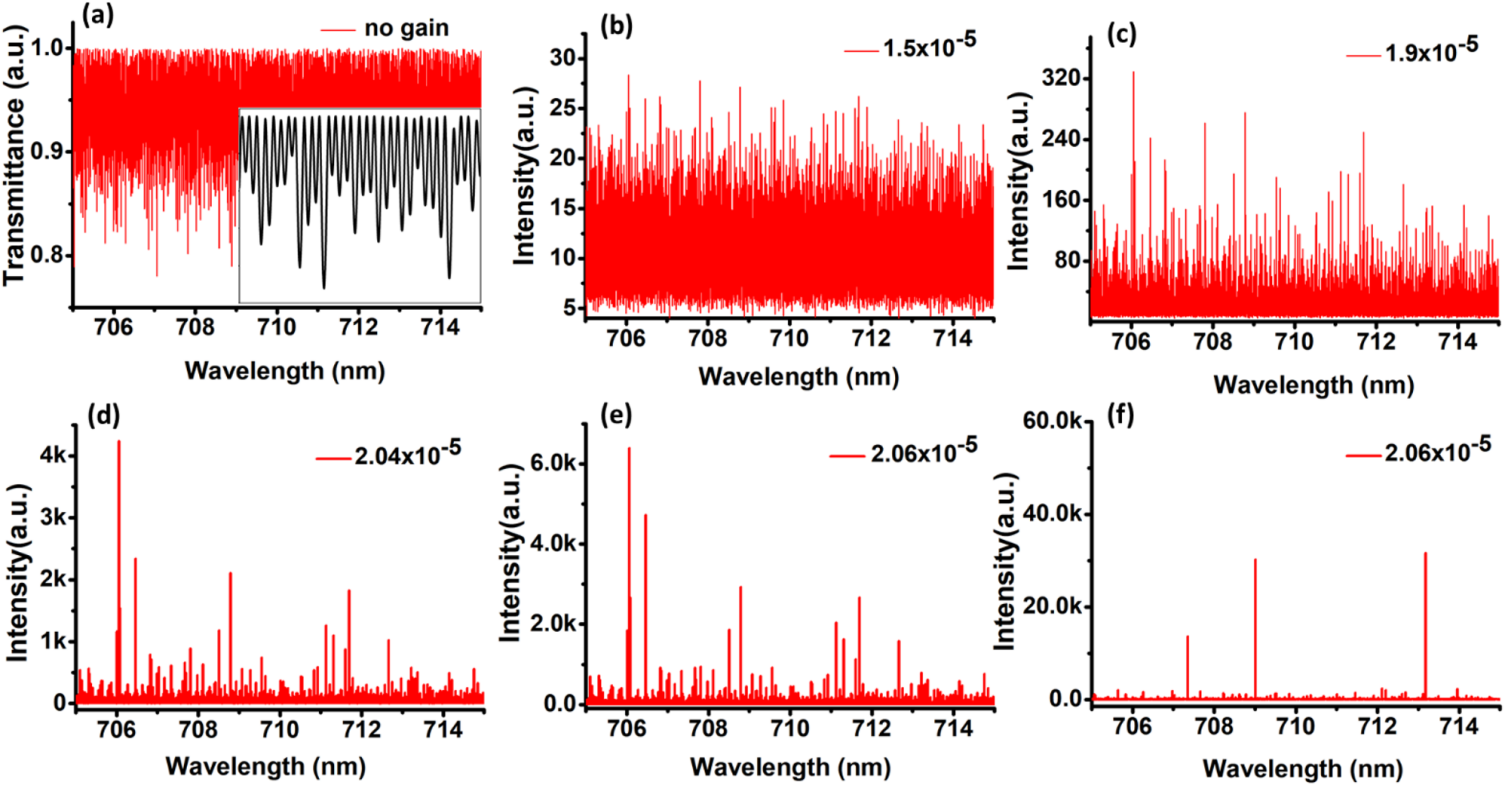
Transmission spectra of the 1D multilayer structure in the presence of disorder in the sizes of the droplets and the inter-droplet separation, for an introduced gain n_2_ = (**a**) zero (**b**) 1.5 × 10^−5^ (**c**)1.9 × 10^−5^ (**d**) 2.04 × 10^−5^ (**e**) 2.06 × 10^−5^. An enlarged image of the spectrum within a wavelength range of 0.4 nm is shown in the inset of (**a**). (**f**) Transmission spectrum of the 1D multilayer structure for another configuration of the same disorder strength and gain n_2_ = 2.06 × 10^−5^.

To substantiate this, we have analyzed the transmission spectra corresponding to the generated droplet array using the 1D transfer matrix method^33^. This is reasonable because the size of the droplets in the generated array is much higher than the wavelength of light. As a result, the collective modes experience only planar boundaries along the axis of the array, reducing the experimental droplet array equivalent to a 1D multilayer structure with gain and disorder in the sizes and inter-droplet spacing. 20 unit cells of the droplets are considered to generate the transmission spectrum, with their sizes and refractive index matching the experimental values. Optical gain is introduced to the multilayers representing the asymmetric droplet by adding an imaginary component to the respective refractive index ($n_{1} + i n_{2}$). The calculation is performed for a wavelength range from 705 to 715 nm, complimenting the higher gain region of the acceptor emission profile. The sizes of multilayers representing the droplets are measured to have a disorder strength varying between 1 and 2 μm based on the experimental findings, and that of MO is measured to be varying between 300 and 500 nm. These disorder strengths are calculated using the pixel to length conversion method as mentioned previously, by analyzing over 50 images of the droplet array. The transmission spectrum of the droplet array with an instantaneous disorder between the aforementioned ranges, in the absence of any gain is shown in Fig. 7a. An enlarged image of the spectrum within a wavelength range of 0.4 nm is shown in the inset. When a small gain is given to such a 1D system, the modes supported by them starts to acquire an increment in intensity. The transmitted intensity profile for a gain $n_{2} = 1.5 \times 10^{ - 5}$ is shown in Fig. 7b. With a gradual increment in the gain, the modes having higher quality factors grows rapidly than others as can be seen from Fig. 7c, designating the lasing. Such lasing of few high-quality modes at a given gain $n_{2} = 2.04 \times 10^{ - 5}$ is shown in Fig. 7d. The mode with the highest quality factor will have the least lasing threshold and realize lasing first. Accordingly, the mode at λ = 706 nm is called the first lasing mode within the considered wavelength region. In addition to this, modes having a closer quality factor to the first lasing mode also may enter the lasing regime, but lases with less intensity than the former. In this sense, the mode at λ = 706.5 nm is identified as the second lasing mode. Further increment in the gain to $n_{2} = 2.06 \times 10^{ - 5}$ results in the gradual increment in emission intensity of the lasing modes as depicted in Fig. 7e. Thus subsequent increment in the gain leads to the coexistence of many lasing modes, resulting in an emission spectrum characterized with multiple lasing peaks. Figure 7f represents the occurrence of lasing modes for another instantaneous disorder at a gain $n_{2} = 2.06 \times 10^{ - 5} .$ The wavelength of the emission, as well as the number of peaks, are fluctuating corresponding to the instantaneous disorder as can be seen from Fig. 7e,f.

Experimentally, the pump pulses experience different array configurations each time due to its intrinsic disorder in the sizes and inter-droplet separation. Hence, as can be seen from Fig. 2d, the collective modes will show fluctuations in the intensity and wavelength from pulse to pulse since they are sensitive to the instantaneous configuration of the dynamic array. Even though the zig zag droplet arrays are also periodic and 1D in nature, the variation in refractive index is not along the axis of the array hence does not provide any feedback to the dye molecules pertaining to the array structure. Hence the collective emission from these arrays is simply the additive intensity of the participating dye molecules and is restricted only to the fluorescence or ASE regime as can be seen from Fig. 2g.

## Conclusion

The proposed linear periodic array of non-WGM droplets with unconventional shapes and intrinsic disorder is effective in providing efficient random lasing emission from the acceptor dyes with reduced lasing thresholds. The design of the array allows the complete radiative transfer of the donor energy to the acceptor, which otherwise is impossible by ordinary methods. The directionality in the emission from the microdroplets lacking any WGM based cavity property can be utilized in droplet mediated light guiding in on chip platforms. Precision control of the disorder strength and its effect on the random lasing generation in the microfluidic platform is still an open area to be explored. The ability to switch on and switch off the WGM lasing in microfluidic platforms offers new ways to explore the behaviour of coupled systems of microdroplets under different experimental conditions. Also, the generation of highly stable microdroplets with excellent shape controllability opens up new opportunities to investigate the WGM lasing properties of structures with unique shapes.

## Supplementary Information

Supplementary Information 1.

Supplementary Information 2.

## Data Availability

The data that support the findings of this study are available from the corresponding author upon reasonable request.
