# Supplementary material for: On chip random lasing performance of the acceptor dye in a specially designed linear and zig zag array of microdroplets with intrinsic disorder
Source: Sci Rep. 2022 Mar 10;12:3939. doi: 10.1038/s41598-022-07104-8 (PMC8913607; doi:10.1038/s41598-022-07104-8)

**Supplement 1**

**On chip random lasing performance of the acceptor dye in a specially designed linear and zig zag array of microdroplets with intrinsic disorder**

**High-Quality Coupled Modes in Microdisc Array**

The whispering gallery modes (WGMs) supported by a micro disc of diameter d=1.2 µm and refractive index n=2.3 in the background of air is simulated using FDTD and the resultant spectrum is shown in Fig. S1 (a). When an array of micro discs are formed, new modes called the coupled modes have emerged in the system in addition to the WGMs. The quality and wavelength of such modes vary depending on the coupling strength between the individual micro discs. Fig. S1(b), S1(c) and S1(d) show the emergence of coupled modes in an array of 10 micro discs for an inter-disc spacing of 0 nm, 300 nm and 600 nm respectively. The quality of the coupled modes decreases with a decrease in the coupling strength as can be seen from the figures. Hence in the weak coupling regime with an inter-disc spacing of 600 nm, the resultant spectra from an array of discs are mostly identical to the single-disc WGMs spectral profile with few survived coupled modes, as can be seen from Fig. S1 (d). The spectrum in magenta color is the single disc WGM profile given for comparison. All the WGM peaks in the array of discs are exactly overlapping with that of the single disc. In addition, irrespective of the coupling strength, the WGMs possess high quality factor compared to the coupled modes. Hence when the gain is implemented, the WGMs are expected to lase.

**Figure S1. (a)** The WGMs supported by a single micro disc. The diameter and refractive index of the disc are d= 1.2 µm and n= 2.3 respectively. The spectral profile corresponding to an array of 10 discs with inter-disc spacing of **(b)** 0 nm **(c)** 300 nm and **(d)** 600 nm respectively. The spectrum in magenta color is the single disc WGM profile given for comparison.


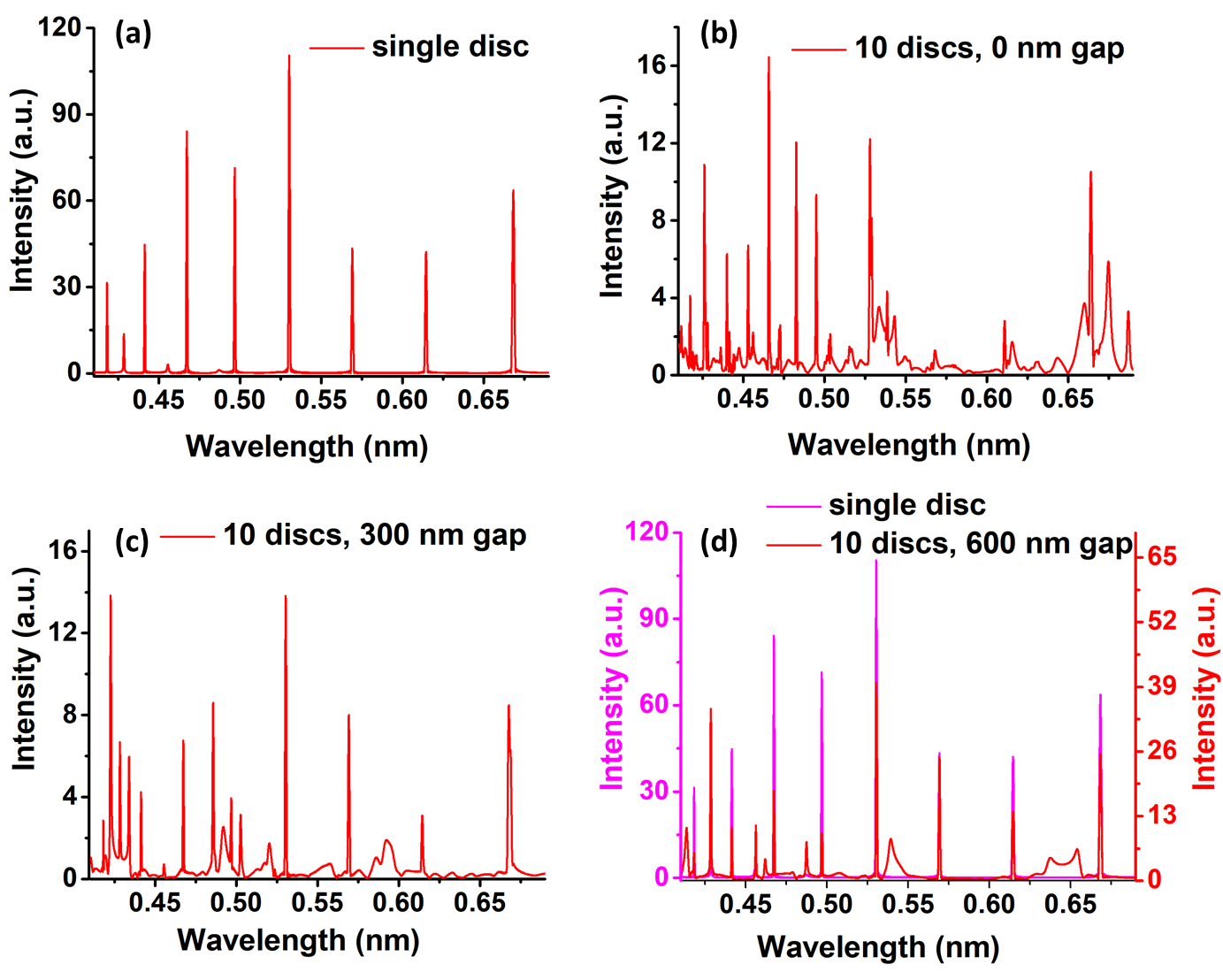


In order to selectively channelize the gain solely in coupled modes, one has to eliminate the WGMs from the system. This can be accomplished in two ways: either by deforming the shape of the micro disc or by increasing the refractive index of the background medium above that of the disc. Keeping the refractive index unaltered, a shape deformed disc is generated by cutting the upper and lower part of a circular disc of diameter ‘d’ and the modes supported by the structure are analyzed. The major diameter of the deformed disc is same as that of the circular disc. The modes formed after introducing a shape deformation to the micro disc is shown in Fig. S2(a) and they differ significantly from the modes in a micro disc as given in Fig. S1(a). The corresponding shape deformed micro disc is shown in Fig. S2(b) and is essentially acting as a Fabry-Perot resonator.

Fabry-Perot resonators are characterized with the expression,

$$FSR=\frac{\lambda^{2}}{2nd}$$

Where, FSR (Free Spectral Range) is the spacing between two modes of a Fabry-Perot resonator of length $d$ and refractive index $n$ at a wavelength$\lambda$.

**Figure S2. (a)** Modes in a deformed disc. The major diameter and refractive index of the disc are d= 1.2 µm and n= 2.3 respectively. **(b)** Schematic image of the deformed disc. **(c)** Modes in an array of 10 coupled deformed discs. The inter-disc separation is 600 nm. **(d)** Comparison of the modes in an array of 10 non-deformed discs (blue) and deformed discs (green), separated by 600 nm gap.


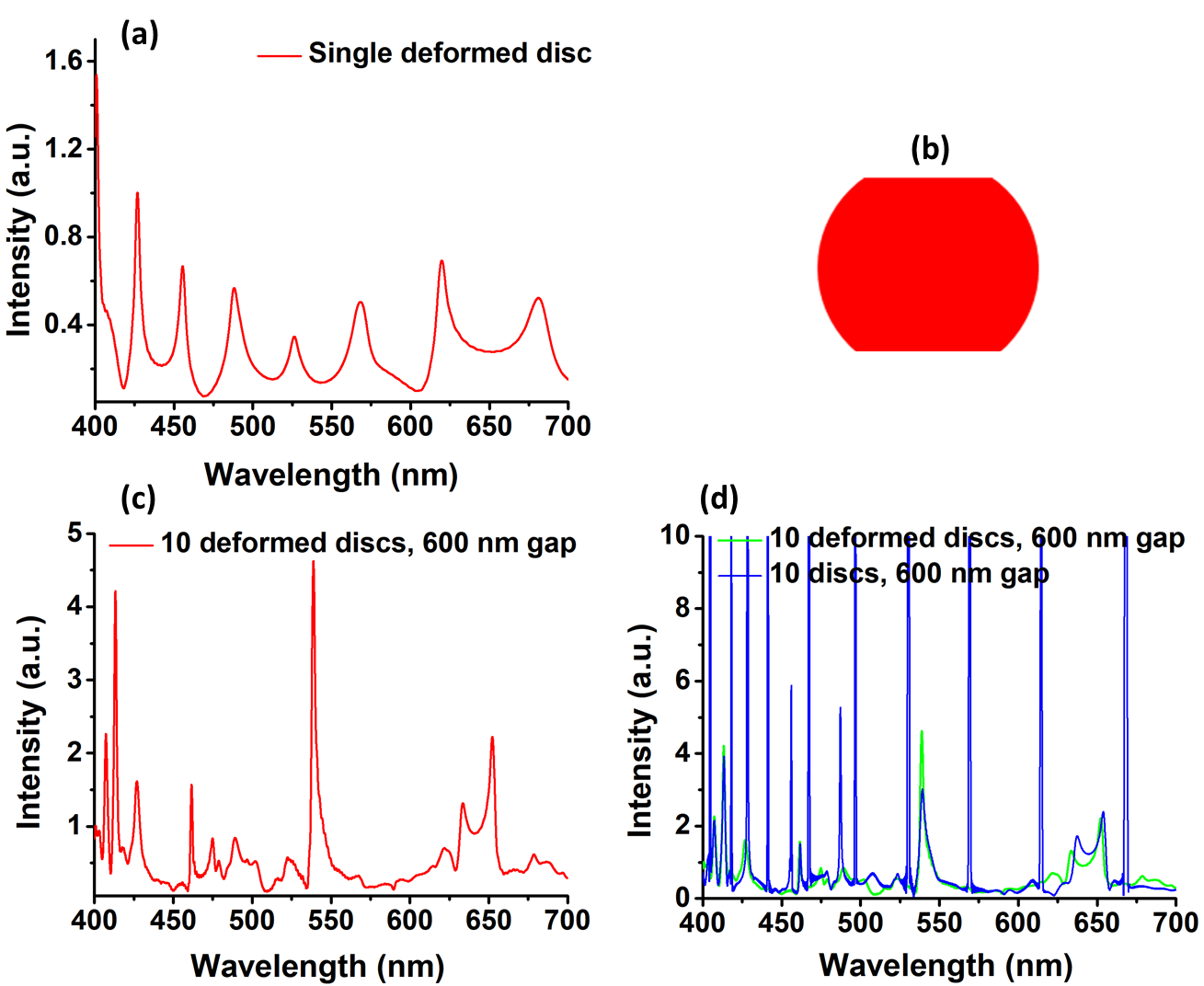


For the deformed disc under consideration, the major diameter,$d=1.2 \mu m$ and$n=2.3$. At a wavelength$\lambda=440 nm$, FSR= 35 nm. From the spectral profile given in Fig. S2(a), FSR= 30 nm, nearly matching with the calculated value. The discrepancy in the values can be attributed to the non-uniform length of the deformed disc consequent to its bi-convex geometry. In an array of deformed micro discs, the resultant modes are thus formed consequent to the interaction between the individual cavity modes. The spectral profile corresponding to an array of 10 deformed discs is shown Fig. S2(c).

Interestingly, when the spectral profiles pertaining to the array of micro discs with and without shape deformation are compared, we have observed that the coupled modes in the array of 10 non-deformed discs and the resultant modes in the array of deformed discs are exactly overlapping, as shown in Fig.S2 (d). This indicates that the new modes called the coupled modes emerged in the array of micro discs as shown in Fig. S1 (d) are nothing but the coupled Fabry-Perot cavity modes pertaining to the structure of the array. The occurrence of such modes is completely independent on the existence or non-existence of the WGMs in the structure.

In order to realize lasing in the collective modes, the quality factor of the modes must be improved, in addition to providing the gain. This can be achieved by increasing the size of the deformed disc. Figure S3 compares the quality of the modes formed in an array of 10 deformed discs each of size 1.2 $\mu m$ and 3 $\mu m$ respectively. As can be seen from the figures, more high-quality modes arise with the increment in the major diameter of the individual disc. These modes can realize lasing in the presence of enough gain.

These simulations provide an insight to realize efficient lasing even without relying on the well-established WGMs. The results are applied to the array of microdroplets generated experimentally with eliminated WGMs. The microdroplets are made large enough to improve the quality of the modes pertaining to the structure of the droplet array. In the presence of gain, such modes can realize lasing.

**Figure S3.** Modes supported by an array of 10 deformed discs of major diameter d=1.2 µm (blue) and d= 3 µm (red). The inter-disc spacing is 600 nm.


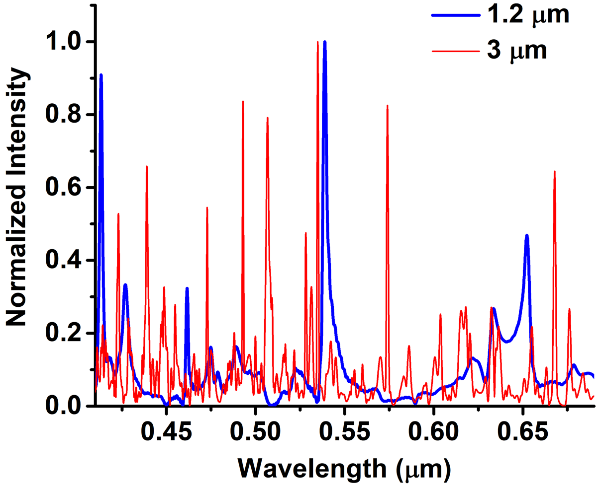

Supplement: Supplementary file 1 — Supplementary Information 1. [file 41598_2022_7104_MOESM1_ESM.docx]
