# Supplementary material for: On chip random lasing performance of the acceptor dye in a specially designed linear and zig zag array of microdroplets with intrinsic disorder
Source: Sci Rep. 2022 Mar 10;12:3939. doi: 10.1038/s41598-022-07104-8 (PMC8913607; doi:10.1038/s41598-022-07104-8)

**Supplement 2**

**On chip random lasing performance of the acceptor dye in a specially designed linear and zig zag array of microdroplets with intrinsic disorder**

**Generation of Microdroplets**

The on-chip dynamic microdroplets are generated using a microfluidic assembly equipped with microfluidic pumps, microfluidic chip and a high-speed digital microscope, purchased from Dolomite Microfluidics. The image of the microfluidic pump and the chip are given in Fig. S1(a) and S1(b) respectively. The channel of the microfluidic chip consists of an X-junction and a T-junction as shown in Fig. S1(b). The X-junction can be fed with three inputs and the T-junction, with two inputs. We have generated the microdroplets using the X-junction of the chip. An image of the X-junction under the microscope is shown in Fig. S1(c). Out of the three inlets to the X-junction, at least one must be supplied with oil, also called the carrier to generate the droplets. The channels of the microfluidic chip we use are internally coated with a layer of PDMS, a hydrophobic material, which enables the generation of solvent droplets backed by the carrier. The inert gas supply essentially provides the pressure to flow the fluids through the channels of the chip and can be controlled using microfluidic pumps. Controlling the pressure using the pumps essentially control the flow rate of the fluids through the channel. When the solvent and the carrier meet at the junction of the microfluidic chip, highly stable droplets of impressive size and shape controllability are formed depending on the flow rate and surface tension of the participating fluids and they flow through the outlet channel of the chip. The imaging of the microdroplets is realized using a high speed digital microscope equipped with high power LED, especially designed for microfluidics (Not shown).

**Figure S1.** Image of the **(a)** microfluidic pump **(b)** microfluidic chip **(c)** X-junction of the microfluidic chip under microscope **(d)** Schematic of the X-junction of the microfluidic chip with the linear array of microdroplets flowing through it.


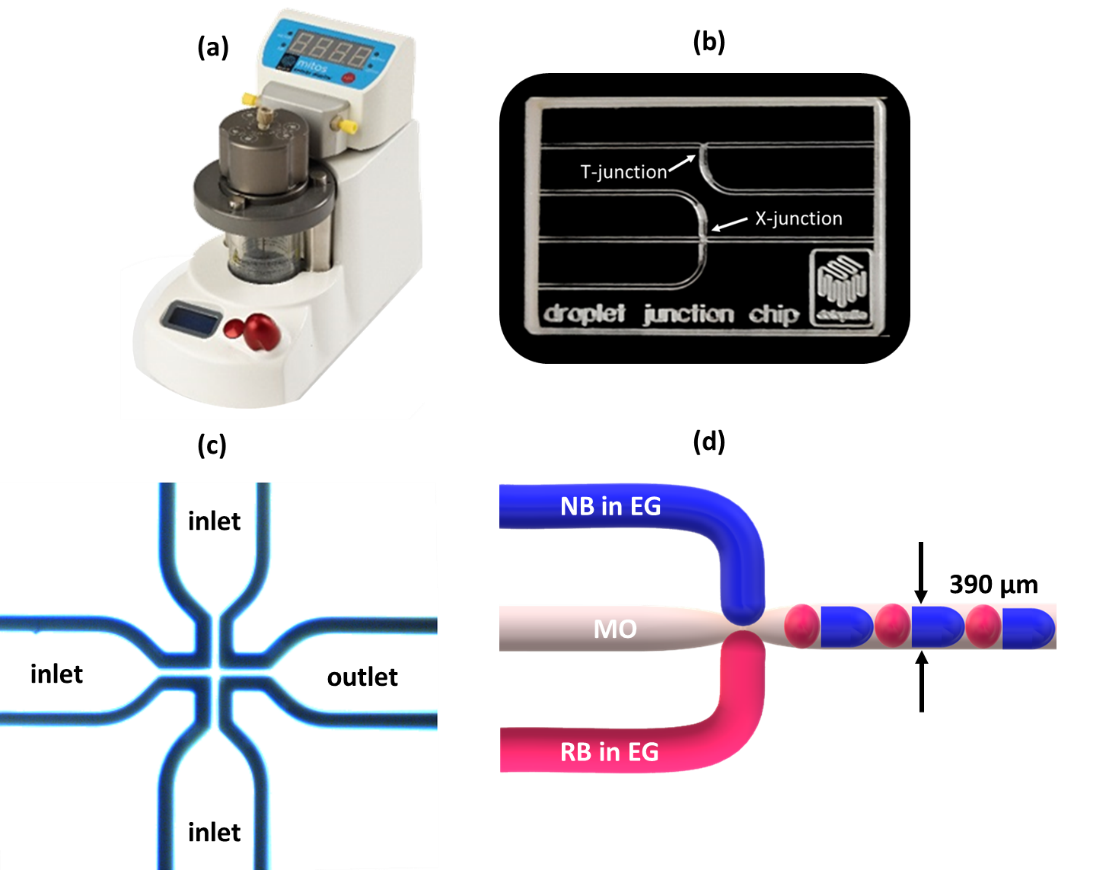


We have used Nile blue (NB) doped Ethelene glycol (EG) as the acceptor and Rhodamine B (RB) doped EG as the donor solutions and mineral oil (MO) as the carrier to generate the droplets. Pure ethylene glycol without doping any dye is used to generate the passive droplets. The solvents and the carrier are fed to the three inlets of the X-junction and carefully adjusted their flow rates to realize the droplet configurations. The zig-zag array of spherical microdroplets is formed when the solvents are pumped with a pressure of 90 mbar and the carrier, with 300 mbar. The triangular microdroplet array can be generated corresponding to a solvent pressure of 120 mbar and carrier pressure of 100 mbar. Once the triangular droplet array is formed, the linear array of droplets can be generated by increasing the pressure of the NB doped EG solution gradually in steps of 10 mbar, keeping the pressures of the RB doped EG solution and MO unchanged. In this way, we can effectively control the length of the acceptor doped droplets without disturbing the size or shape of the donor (or passive) droplets. A schematic of the channel of the microfluidic chip with the linear microdroplets flowing through it is presented in Fig. S1(d).

**Experimental Setup**

Figure S2(a) shows the schematic of the experimental setup. The components are (1) Nd-YAG laser (2) Neutral density filter (3) Cylindrical lens (4) Slit (5) Microfluidic chip (6) Microfluidic pumps (7) Plano-convex lens (8) Fiber coupled spectrometer (9)CCD. Generated microdroplets are excited using a Nd-YAG laser of 6 ns duration, emitting at 532 nm. The pump beam is made into a stripe using a cylindrical lens of 20 cm focal length. The slit width is maintained at 7mm throughout the experiment. The emission is collected perpendicular to the pump beam using a fiber-coupled spectrometer (Ocean Optics Maya-Pro 2000) and the imaging of the pump illuminated droplet array is done using a colored CCD. A photograph of the experimental setup is shown in Fig. S2(b).

**Figure S2. (a)** Schematic of the experimental set-up. The components are (1) Nd-YAG laser (2) Neutral density filter (3) Cylindrical lens (4) Slit (5) Microfluidic chip (6) Microfluidic pumps (7) Plano convex lens (8) Fiber coupled spectrometer (9) CCD. **(b)** Photograph of the experimental set-up.


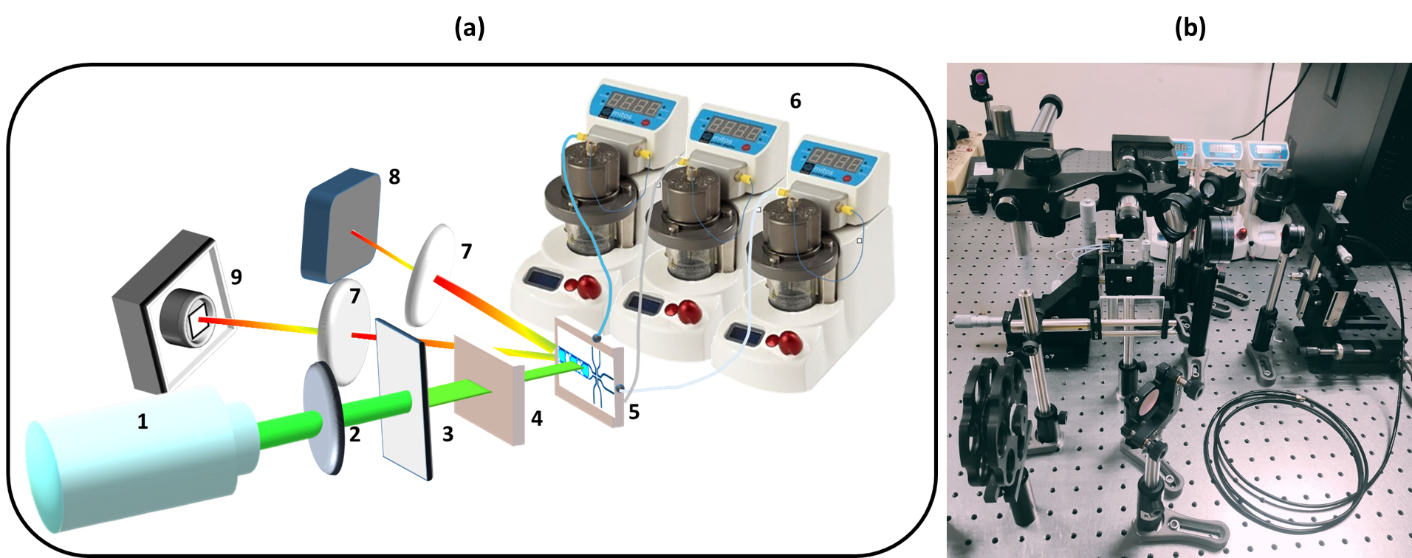

Supplement: Supplementary file 2 — Supplementary Information 2. [file 41598_2022_7104_MOESM2_ESM.docx]
